# Talking about death and dying in a hospital setting - a qualitative study of the wishes for end-of-life conversations from the perspective of patients and spouses

**DOI:** 10.1186/s12904-020-00675-1

**Published:** 2020-11-02

**Authors:** Heidi Bergenholtz, Malene Missel, Helle Timm

**Affiliations:** 1 Holbaek Hospital, Region Zealand, Smedelundsgade 60, 4300 Holbæk, Denmark; 2 REHPA, Knowledge Centre for Rehabilitation and Palliative Care, National Institute of Public Health, University of Southern Denmark, Vestergade 17, 5800 Nyborg, Denmark; 3 Rigshospitalet, Blegdamsvej 9, 2000 Kbh.Ø, Denmark

**Keywords:** Palliative care, Communication, End-of-life, Hospital, Patient, Spouses

## Abstract

**Background:**

End-of-life (EOL) conversations are highly important for patients living with life-threatening diseases and for their relatives. Talking about the EOL is associated with reduced costs and better quality of care in the final weeks of life. However, there is therefore a need for further clarification of the actual wishes of patients and their relatives concerning EOL conversations in an acute hospital setting.

**Aim:**

The purpose of this study was to explore the wishes of patients and their relatives with regard to talking about the EOL in an acute hospital setting when living with a life-threatening disease.

**Methods:**

This study is a qualitative study using semi-structured in-depth interviews. A total of 17 respondents (11 patients and six spouses) participated. The patients were identified by the medical staff in a medical and surgical ward using SPICT™. The interview questions were focused on the respondents’ thoughts on and wishes about their future lives, as well as on their wishes regarding talking about the EOL in a hospital setting.

**Results:**

This study revealed that the wish to talk about the EOL differed widely between respondents. Impairment to the patients’ everyday lives received the main focus, whereas talking about EOL was secondary. Conversations on EOL were an individual matter and ranged from not wanting to think about the EOL, to being ready to plan the funeral and expecting the healthcare professionals to be very open about the EOL. The conversations thus varied between superficial communication and crossing boundaries.

**Conclusion:**

The wish to talk about the EOL in an acute hospital setting is an individual matter and great diversity exists. This individualistic stance requires the development of conversational tools that can assist both the patients and the relatives who wish to have an EOL conversation and those who do not. At the same time, staff should be trained in initiating and facilitating EOL discussions.

**Supplementary Information:**

**Supplementary information** accompanies this paper at 10.1186/s12904-020-00675-1.

## Background

Many people in today’s ageing population live with life-threatening chronic diseases for several years [1]. Therefore, it is crucial to understand at what point it is time to talk about the end-of-life (EOL) and how. Research has shown that talking about and planning the EOL is important for how the final days in a patient’s life may play out and is associated with reduced costs as well as a higher quality of care in the final weeks of life [2–4]. A recent Danish study has even shown that patients engaged in EOL planning have a significantly increased life expectancy [5]. As many patients are hospitalized during the final year(s) of life [6], the EOL also needs to be addressed in hospitals. Patients living with a life-threatening disease express readiness to talk about the EOL before the healthcare professionals (HPs) address the issue [7], and these conversations take place very close to death, in the last week of life [8]. Thus, although the patients have thoughts about the EOL, the communication and documentation of their wishes are inadequate [9].

The barriers in initiating EOL discussions are well described. These are, for example, lack of competencies of HPs [10]; underestimating the need for information [11]; and the fear of taking away the patient’s hope [12]. This may result in a late initiation of EOL conversations. A study has shown that for half the patients with chronic end-stage diseases, who have lived with those diseases for many years, their wishes regarding the EOL were not met [13]. When talking about the timing of the conversations, Mack et al. showed that EOL conversations were only documented for 27% of patients and often took place during acute care in the hospitals late in the illness trajectory [14]. This means that many patients may be hospitalized (and even die) without having talked about their wishes for the EOL [15, 16]. Furthermore, it is worth mentioning that death and dying are still taboo topics in many cultures, which may also serve as an explanation for why EOL conversations are avoided.

From a family viewpoint, patients and relatives may not always share the same perspectives on a “good death” [17] and the relatives are described as having unmet needs regarding communication in hospital settings [18].

For the purpose of this study, ‘EOL conversations’ refer to the broad concept of conversations held between HPs, patients and their relatives concerning thoughts, wishes and planning of the EOL. Initiatives such as Advance Care Planning (ACP) [19] are often used and referred to when talking to patients about the EOL, as they entail decisions concerning treatment level and preferred place of death. There is evidence that ACP impacts positively on the quality of the EOL [20], particularly in nursing homes, where it is seen that ACP reduces the rate of hospitalisation among nursing home residents [21]. However, ACP initiatives are not systematically implemented in the Danish healthcare system [22], so it remains relatively unknown how EOL matters are addressed in acute hospital settings in Denmark. One study has shown that the topics addressed in EOL conversations in hospitals are limited to “the here-and-now”, decisions about the level of treatment and to a lesser extent the patients’ and relatives’ needs to share their thoughts and wishes concerning the EOL when the patient is hospitalised [23]. This is despite the existence of national recommendations regarding EOL conversations in Denmark [24, 25] that highlight the importance of addressing the EOL early in a disease trajectory where the patient is still able to make decisions about future treatment and care.

Therefore, the aim of the study is to explore patients’ and their spouses’ wishes regarding EOL conversations in an acute hospital setting when living with life-threatening diseases.

## Methods

This is a qualitative study using semi-structured, in-depth interviews to explore patients’ and relatives’ wishes regarding talking about the EOL. Using semi-structured interviews as a method is based on the understanding that patients’ and relatives’ life experiences are essential in understanding their actual illness and life situation [26].

### Sample and data collection

The study took place in a Danish acute care hospital with 338 beds and covering 1298 deaths per year. Two separate inpatient departments were selected: a general medical department covering 10 units and an acute surgical abdominal department covering one unit. The medical department had 165 beds and included cardiology, nephrology (also dialysis), geriatrics and pulmonary medicine. The surgical department had 38 beds (26 acute and 12 electives) and the patients ranged from acute abdominal and gynaecological diseases to cancer patients who could not be offered any curative treatment.

This selection was chosen because of the opportunity to include patients who had various diagnoses, including malignancies as well as chronic life-threatening diseases. In the period March 2019 – June 2019 a total of 17 respondents (11 patients and six spouses) with various life-threatening diagnoses participated in the study (Table 1).

**Table 1:** Respondents

Respondent Patient (P) Spouse (S)	Sex	Age	Diagnosis	Marital status	Interview location (home or hospital)
1 (P)	F	60–69	Liver cirrhosis	Lives alone	Hospital
2 (P)	M	40–49	Liver cirrhosis	Lives alone	Hospital
3 (S)	M	50–59	Pancreatic cancer (wife)	Married	Hospital
4 (P)	M	50–59	Liver cirrhosis	Lives with girlfriend	Hospital
5 (P)	F	70–79	COPD	Married	At home
6 (S) to 5	M	70–79	COPD (wife)	Married	At home
7 (S) to 8	F	60–69	Renal failure (dialysis) (husband)	Married	At home
8 (P)	M	60–69	Renal failure (dialysis)	Married	At home
9 (P)	F	80–89	Leukemia (dialysis)	Married	At home
10 (S) to 9	M	80–89	Leukemia (wife)	Married	At home
11 (P)	M	70–79	Renal failure (dialysis)	Married	At home
12 (P)	M	70–79	Heartfailure	Married	At home
13 (S) to 12	F	60–69	Heart failure (husband)	Married	At home
14 (P)	M	60–69	Cancer (colon)	Single	Hospital
15 (P)	F	70–79	Cancer (lung)	Married	Hospital
16 (P)	M	60–69	Heart failure	Married	At home
17 (S) to 16	F	60–69	Heart failure (husband)	Married	At home

The uneven number was due to the fact that not all of the patients had a relative they wished to include, and not all of the relatives wished to participate. In one case (respondent 3) the patient did not wish to participate but the spouse accepted. It was not a criterion for the study that the relative should be a spouse, merely that they should be a person with whom the patient had a close relationship. This person was indicated by the patient. It was a coincidence, therefore, that all participating relatives were spouses. The patients were identified by SPICT™ (Supportive & Palliative Care Indicator Tool) [27] to ensure that the study included patients who were in a palliative phase of their disease trajectory. We were aware that the patients and their relatives may not have talked to HPs about death and dying during hospitalization, since, as described in the background section, not everybody is offered an EOL conversation. However, it was not a criterion to have had this conversation, as our aim was to explore the need and wish to address the EOL in a hospital setting (regardless of experiences). The identification was made by a nurse who was trained in using SPICT™ in each department. In order to be included in the study, a minimum of two of the general indicators and one of the clinical indicators had to be fulfilled (a positive SPICT™). The general indicators referred to generally poor or deteriorating health, whereas the clinical indicators referred to one or multiple life-limiting conditions. The relative/spouse was chosen by the patient as a person whom they saw as being close to them and who might also be affected by their situation in living with a life-threatening disease.

The patients and spouses were identified during a hospital stay and were offered the opportunity to be interviewed either at the hospital or at home after discharge. If the interview took place in the hospital, a private room was used to maintain anonymity and confidentiality. If home was preferred, then the nurses (or researcher) would schedule a suitable time.

Four nurses (three from the medical ward and one from the surgical ward) were trained by the first author in conducting interviews. The nurses were not directly involved in the care of the patients they interviewed; when they conducted the interviews they wore their own clothes (not a uniform), signalling that the interview was not part of and would not affect their treatment in the hospital. The nurses and the researcher (first author who was an experienced interviewer and also trained as a nurse) conducted between two and four interviews each, and the researcher functioned as a supervisor for the nurses. It was the job of the trained researchers (authors) to ensure that COREQ guidelines were followed [28]. This meant that it was the task of the researchers to ensure the scientific quality of the study.

The interviewers guided the interview by focusing openly on the patient’s life situation; their thoughts on and wishes for their life as well as their experiences of talking about the EOL (if this was addressed by the respondents). The interview guide was developed for this present study. All interviews began with an open question: “Would it be ok if we talked about your (and your spouse’s) life with a serious disease – could you tell me what it has meant/means to be living with this disease?”. Subsequently there were questions such as “What thoughts have you had about the future?”, “What is important for you to talk to the HPs about when you (your spouse) were hospitalised?”, and (if addressed) “What thoughts have you had about the final days of your (your spouse’s) life?”, “What gives you meaning and hope?” and “Who do you prefer to talk to about this?”

The interviews were consequently guided by questions such as “Could you tell me more?” and “What did that mean to you?” in order to further explore the stories and situations described by the respondents.

The interviews were recorded, transcribed verbatim afterwards and lasted between 23 and 56 min (mean 40 min).

### Analysis

The analysis of the interviews followed Creswell’s five-step data analysis for qualitative inquiry [26] (Table 2). The analysis was led by the first author, who has previous experience in qualitative research as well as in palliative care. The findings were discussed among all authors (all trained researchers) as well as the nurses from the departments who participated in the research process. The analysis progressed from an initial reading of all transcripts of the interviews, to organising and searching for themes and patterns, to the identification of the meaning in each interview as well as across all stories concerning the aim of the study.

**Table 2:** Steps of data analysis (Creswell [26])

Managing and organizing data	The transcripts of the interviews were printed for each participant and the data was stored securely in a locked room. The analysis mode was chosen, and was first done by hand and secondly entered in a Word document.
Reading and memoing emergent ideas	All transcripts were read several times by the first author in order to obtain a sense of the interview as a whole. Initial thought and codes were written in the margins of the transcripts.
Describing and classifying codes into themes	A search for patterns was initiated. The initial codes were named and classified into themes, and were related to the phenomena of interest (research question). This was done by focusing on both unique and recurring themes across the interviews.
Developing and assessing interpretations	The meaning of each interview/story was identified, and even though each interview was unique there were themes which recurred. The codes were interpreted and discussed by the authors.
Representing and visualizing the data	The meaning of the interviews was presented, and is represented in the findings section. Excerpts will be presented in this section to illustrate the themes and to allow the reader to judge transferability.

### Ethical considerations

The Danish Data Protection Agency registered the study (REG-163-2017) and the storage of data was followed accordingly. Furthermore, the Declaration of Helsinki was followed and the respondents were informed both verbally and in writing about the project and their participation. They were guaranteed anonymity and confidentiality, and they provided their consent when the interview was arranged and again before the interview started.

When the respondents were approached and recruited they were in a vulnerable state, as the patients were hospitalised due to deterioration or acute disease. The interviewer and the HPs in the individual department collaborated to find the right time to inform the patient, i.e. when they were not in an acutely deteriorating state. It is important to note that the patients might not have had a conversation about the EOL in the hospital. This meant that the respondents might not have talked about their disease as life-threatening or about being in need of palliative care. It was therefore considered unethical if it was revealed during the interviews that the patient was identified as having palliative care needs (by being identified by SPICT™). The interviewer did not mention the words ‘life-threatening’, ‘death’ or ‘dying’ unless the patient and spouse mentioned it.

### Findings

Two themes were identified during the analysis with regard to how the respondents wished to talk about the EOL in an acute hospital setting. The first theme relates to the respondent’s reflections on life, death and dying and the wish to talk about this in general, as well as their hopes when living with a life-threatening disease, whereas the second theme describes the respondent’s wish to talk about the EOL in an acute hospital setting and how they see the role of the HPs in this context.

### Theme 1: End-of-life as an individual matter - reflections on death and dying when living with a life-threatening disease

The lives of both the patients and their spouses were marked by their disease. Respondents expressed how very different their lives were, in comparison to before the onset of disease. The loss of work, friends and physical abilities was frustrating for the respondents. The general need and wish to both talk and think about the EOL seemed secondary for the patients; the impairment to their everyday lives received all the focus. The respondents were concerned about the prospect of becoming dependent on others by exacerbating the illness. The thought of being dependent on others eclipsed all other thoughts for the patients, and they described how they would rather die than end up in such a situation. Stories of fear of dependency on others were a pervasive theme when talking about the progression of the disease.

*Interviewer: “When you say” much worse, “what do you mean by that?”*

*Respondent 12 (Patient):“… I'd rather fall down dead. You've got to be reliant on so many people.”*

*“I’ve decided that if my life is going to be hell, I’d rather not be here at all.” (Patient- Respondent 16)*

Even though the primary concerns for the respondents were about everyday life when living with a life threatening disease, their wishes and needs for EOL conversations were also addressed. However, these varied widely between the respondents. It was an individual matter, ranging from not wanting to talk or think about the EOL at all to wanting to plan the EOL in detail.

At one extreme were the respondents who had many considerations about death and dying, and who had also discussed these with their spouses and other relatives. They had planned everything in a way that would take care of the bereaved relatives when the patients themselves were no longer able to do so:

*“The most important thing for me is that when I’m no longer here, my daughter will have to look after herself. It’s hard to accept that I won’t be here and will no longer be able to help.” (Patient - Respondent 4)*

For this group of patients (who wished to address the EOL), it involved decision-making concerning legal wills and ensuring that their relatives were financially secure, as well as deciding on do-not-resuscitate (DNR) orders. For some, it also involved detailed planning of how they wanted to leave this world, including choosing a coffin or making other plans for after they had died.

At the other extreme, the respondents did not wish to spend time on thinking and worrying about the EOL:

*“… but when that day comes, and it will, you have to say goodbye... but I don’t want to think about that all my life.” (Patient - Respondent 9)*

A distant view of the EOL was also expressed through a more optimistic approach, explaining that the respondents thought it was about being positive and accepting – a way to get as much out of the rest of their lives as possible:

*“You have to try to be a bit positive, there’s no use sitting in a corner blubbering because that’s no help. It won’t change anything, so I think you’ve got to stay positive and see the good things.” (Spouse- Respondent 7)*

Some patients were not at the same stage as their spouse regarding the need to talk about the EOL. One couple, for example, had very different approaches to talking about death and dying. The patient (a male) was very open and had a religious approach to where he would go after death, whereas his wife did not want to discuss this matter with him:

*“...my wife doesn’t think so much about it. She sticks her head in the sand, hoping it’ll go away. But, of course, it won’t go away.” (Patient -Respondent 16)*

This difference created some challenges as it left the patient isolated with his wish to talk about the EOL.

The concept of hope was mentioned several times by the respondents. Hope was expressed as being something to look forward to – something which could happen in the future, when maybe the patients would be in a better state. It was described in various ways, as some hoped for an actual cure and survival, while others hoped that their physical state and energy levels would improve to allow them to engage in more activities:

*“I know very well that right now she’s incurably sick, but the only thing you can do is keep her alive … and hope that one day they find some sort of cure, isn’t it?” (Spouse-Respondent 3)*

*“(when I get better) I’ll have bought myself a little motorboat, so I’ll go out and catch some cod and things, and enjoy myself … just enjoy life.” (Patient-Respondent 14)*

Some also found that they took comfort and hope from the doctors continuing to offer treatment:

*“there are some that say … as long as your heart’s beating we can treat you, and that’s really reassuring.” (Spouse-Respondent 3)*

This theme showed that the respondents’ approaches to thinking and talking about the EOL come with a wide range of individual wishes and perspectives; this wide range was the case for both the patients and their spouses.

### Theme 2: In between crossing boundaries and superficial communication – expectations on talking about end of life with the health care professionals

The wide range between the respondents described in theme 1 was also the case when looking at the respondents’ wishes to talk about the EOL with the HPs in the hospital. For some, it was a sensitive and personal matter which they restricted to addressing with family and friends, rather than something they expected to be addressed in a hospital setting. However, at the other end of the continuum were respondents who expected the HPs to be capable of talking about the EOL:

*“ … I expect that the staff can talk about everything … the young ones have no problems talking about it” (Patient-Respondent 4)*

In contrast, some respondents felt that HPs were overstepping the mark if they addressed the EOL in that setting:

*“She was admitted before Christmas. She had a bowel infection, and the doctor said to her ‘have you thought about your funeral yet?’ … I think that was unnecessary and I was annoyed … but they just don’t understand … I think some ethics are needed in all this.”(Spouse-Respondent 3)*

A more ethical approach from the HPs was called for, and respondents expressed a wish that doctors and nurses showed greater sensitivity in talking about the EOL. Another example was related to the location at which EOL discussions were held. This could be the hospital hallway or the patient’s room in the hospital, where there was no privacy:

*“The conversation I had with one of the doctors, we talked out in the hall, he gave her 3 months to live. And then he made it six, and then he said she could well have a year. Then he couldn’t say anymore, and ran into the office.” (Spouse-Respondent 3).*

Furthermore, several respondents mentioned that talking about the EOL was not something that should be done when the patients were alone. The respondents preferred to have family present when addressing sensitive matters. When talking about prognostics, dying was neither something that the patients found likely to happen any time soon, nor something they wished to talk about:

*“I don’t need that, and I feel like if you have a date … if you say there’s half a year left, then you just count down the days, and I don’t think there’s any reason for that.” (Patient-Respondent 8)*

The concept of trust and ‘chemistry’ in the relationship with the HP was mentioned by several respondents. They mostly characterised the HP staff as being nice and meaning well, but also assumed that the HPs did not have the time to talk about the EOL. The roles of being a patient and being a nurse at work were described by some as a barrier to talking about sensitive matters:

*“The hard thing is one’s private life. I know the nurses from three weekly meetings. But firstly they have their readings to take, and then they might just say ‘well that’s done then’, and then they’re finished for the day. There are all these problems I have, and there’s a person who just get up and leaves.” (Patient-Respondent 11)*

The relationship to the individual HP was seen as crucial for establishing trust in communication, but what was expected of the HPs was related to the medical condition and treatment; it was not expected that existential and emotional matters were addressed. Others felt that sensitive matters like death and dying were not something the HPs themselves felt comfortable addressing.

*“The doctors and nurses don’t talk much about personal matters … it’s more the tangible stuff connected to illness, and what you can do to feel better, and what you can’t do, and what options you have … no doctor or nurse asks about the main thing – you have to deal with that yourself.” (Patient-Respondent 16)*

One said that it felt like only superficial matters concerning the disease were addressed by the HPs:

*“I think they do good solid work, but they don’t always get what you’re talking about … it’s more like they just come with the medicine. They come with food, with this and that … in that way it’s a bit superficial.” (Patient-Respondent 12)*

To sum up, the findings show that the diversity of responses regarding general wishes and needs for talking about the EOL also applies in a hospital setting. A life-threatening disease can be all-encompassing in everyday life and the dominant focus was hope for regaining a better physical state. The decline of physical function had serious consequences for the respondents’ lives, and a wish to ‘get better’ featured highly. It seemed that the desire to talk about the EOL in a hospital setting was defined by the context. The respondents described how the relationship to the HPs in a hospital context was often a more professional rather than personal one, owing to their medical approach.

## Discussion

Living with a life-threatening disease for many years creates many challenges for the patient and their spouse. While everyday life seemed to be the major focus for the respondents in this study, there was also an awareness that the disease would progress, and the patients would be in need of palliative care at some point. Therefore, the findings from this study may point towards ways for palliative care and rehabilitation to work side by side when addressing EOL matters. Rehabilitation can support and improve everyday activities, while the palliative care approach can relieve symptoms related to physical, psychological, existential and psychosocial problems. The link between palliative care and rehabilitation has been the subject of much discussion in recent years, as many patients with life-threatening diseases are living considerably longer; this makes both rehabilitation and palliative care interventions relevant. A recent Danish review shows us that even though there are structural and professional challenges in the coordination of rehabilitation and palliative care, it is meaningful for patients to engage in both efforts throughout a disease trajectory [29]. In our study, respondents wished not only for improved physical function but also to have more energy to engage in social activities. As most rehabilitation initiatives for people with life-threatening diseases consist of physical exercises developed for cancer patients [30], new ways of thinking about the integration of palliative care and rehabilitation might be needed. Future palliative rehabilitation interventions may also be adapted to the individual needs and wishes of the patients and their relatives; this includes an increased focus on EOL conversations, namely when and how to address the topic. The fine line between offering and withdrawing curative treatment was also demonstrated in our study. Some found hope and comfort in the doctors continuing to offer active treatment (hoping for a cure), whereas others were aware of the disease having an endpoint and wished to die before they were further impaired. For an HP to clarify where the patient and their spouse stand requires a certain familiarity with the patient, which can be achieved through conversations. Gerber et al. [31] also described that patients and caregivers are balancing between not knowing enough and knowing too much. It may create a feeling of being unsafe if the patients and their caregivers are hoping for a cure and the HP discloses imminent death. The solution is not simple, but, as Gerber et al. suggest, talking and deciding on the EOL is a contextual, personal, relational, conditional and flexible process that should not be restricted to a single conversation – it requires interdisciplinary collaboration.

The wish to talk about the EOL varied between the respondents in our study. This in accordance with both a study by Simon et al. [32], who found that there is heterogeneity in older people and their caregivers regarding their needs to address EOL issues, and a study by Gerber et al. [33], who found that older patients in particular avoided EOL discussions, as they preferred not to think and talk about dying and consequently left EOL decisions to their family members.

In our study this also created a challenge if the patient and their spouse were not on the same page regarding their desire to talk about the EOL – a lack of EOL conversation could easily leave one of them feeling isolated. It can thus be crucial for HPs to address and maybe even mediate between the patient and spouse in their different EOL approaches. In our study, the respondents’ wishes to talk about EOL issues with the HPs in the hospital seemed to be a balance between superficial communication and crossing boundaries. This means that the balance was between respecting the wishes and boundaries of individual patients and spouses, while at the same time offering a conversation for those in need and not limiting the interaction to superficial matters. To embrace this diversity, while ensuring a trusting relationship in an appropriate physical space, places heavy demands on HPs. This balance could also be described, as done by Simon et al. [34], as “Not yet” and “Just ask”. Factors like timing, location and relationship with the HP are crucial when communicating about the EOL. What we saw in our study was that EOL conversations are offered on what could be called “the premises of the system”. This means that the conversations were offered where and when it suited the HPs, instead of being organised according to the needs of the patients and their spouses. This resulted in some respondents describing how they felt having to talk about the EOL in a hospital hallway or whenever the doctor had the time as overstepping the mark. The respondents felt that the patient should have a relative present. Our study showed that what was expected from the HP was related to the disease and its treatment, rather than an actual EOL conversation. A recent review [35] indicates that the use of personal narratives can relieve psychosocial and existential suffering in a hospital setting, but that a flexible intervention is required that is adjusted to both the setting and individual needs. Findings from this study reveal that the HPs might have to address EOL issues in another way – a way that embraces the position of the patient at that moment, as well as being sensitive to the fact that the patients and their spouses may not be at the same page. This supports findings among nursing home staff [36] that highlight the point that although it is important to initiate EOL conversations, it is also important to be sensitive to the diversity of opinions and the timing of these conversations. Tools like ACP may be too restrictive for some scenarios, and may not be able to take the wishes of the individual patient and their relatives into account [37]. ACP presents a systematic way of engaging in EOL conversations, however, which is crucial for ensuring that all patients are offered the opportunity to share their EOL wishes. Another example is the planning part of SPICT™, which was used for identifying the respondents in our study. Even though SPICT™ is not a conversation tool it implies a planning part that says: *“Agree on a current and future care plan with the person and their family”* [38]. Bearing the findings from our study in mind, it is highly important that this care plan is guided by the patients’ and spouses’ viewpoint; it should be initiated by trained HPs who can master the balance between superficial communication and crossing boundaries while embracing the diverse wishes in talking about the EOL.

### Strengths and limitations

The limitations of this study include partly the multiple interviewers (including nurses as interviewers), and partly the timing of the interviews. Firstly, this study included five interviewers in all. Even though the nurses were fully trained in conducting interviews, their lack of research training might have resulted in different approaches during the interviews. The first author guided them throughout the process, however, and ensured that the study followed the COREQ guidelines [28]. From our perspective, the inclusion of nurses working in clinical practice is also a strength of this study, as this approach was chosen to link clinical practice and research. The nurses gained insight into the lives of patients and relatives by stepping out of their role as nurses and conducting interviews. This meant that they developed a new perspective for talking to patients about the EOL.

We are aware that the fact that nurses conducted the interviews may have led to a response bias. As nurses represent the hospital they may have impacted the respondents’ answers as a result of a power imbalance. However, our experience was quite the opposite. The respondents were satisfied and comforted by the fact that the nurses were familiar with the hospital system and had some knowledge of diseases.

Secondly, the patients and spouses were approached at the hospital during a deterioration. This might have meant that the patients and spouses were preoccupied with the decline that had just occurred and so this took up a lot of the interview time. The question of whether this limitation could have been avoided by waiting a longer period before interviewing is difficult to answer. The fact that they have had the disease for many years, and that the functional decline has lasted for a longer period, might support the idea that it would not have made much of a difference. Furthermore, it should be noted that we found no difference in the responses by participants who were interviewed at home versus in the hospital.

## Conclusion

This study shows that wishes concerning conversations about EOL issues are a personal and individual matter for patients and spouses. Living with a life-threatening disease is challenging, as declining functionality has heavy consequences for everyday life: the fear of further impairment preoccupied the respondents more than thinking about the EOL. There is great diversity, however, in respondents’ wishes concerning EOL conversations; this is also shown in their wishes in engaging in EOL conversations with HPs. Responses ranged went from not wanting to think or talk about the EOL and feeling that HPs were overstepping the mark if they addressed it, to a desire to talk about and plan the EOL in detail with an expectation that the HPs would engage in this.

Our study highlights the need for individualised conversation tools that can embrace diversity and ensure that all patients are systematically offered the possibility to talk about the EOL in a hospital setting– without being forced to do so. Furthermore, staff training to initiate and facilitate EOL discussions is crucial, because a tool on its own will not be able to change clinical practice.

## Supplementary Information

**Additional file 1.** COREQ (COnsolidated criteria for REporting Qualitative research) Checklist.

## Data Availability

The data material used in this study are available from the corresponding author on reasonable request which will not conflict with the anonymity and confidentiality of the data.

## Abbreviations

ACP: Advance Care Planning
EOL: End of life
HP: Healthcare professionals (doctors, nurses and social and healthcare assistants)
